# Relationship between molar incisor hypomineralization, intrapartium medication and illnesses in the first year of life

**DOI:** 10.1038/s41598-022-05628-7

**Published:** 2022-01-31

**Authors:** Emilia Acosta, Olga Cortes, Sonia Guzman, Montserrat Catala, Monica Lorente, Julian Jesus Arense

**Affiliations:** 1 Department of Paediatric Dentistry, University of Murcia, Murcia, Spain; 2 Department of Paediatric Dentistry, University of Valencia, Valencia, Spain; 3 Gynaecology and Obstetrics Section, Santa Lucía University General Hospital, Cartagena, Spain; 4 Department of Public Health Sciences, School of Medicine, University of Murcia, Murcia, Spain

**Keywords:** Diseases, Medical research, Pathogenesis

## Abstract

Molar incisor hypomineralization (MIH) affects the first permanent molars and permanent incisors whose formative embryological process develops around birth and the first year of life. This study’s main objective is to assess the relationship between MIH, on the one hand, with the administration during childbirth of epidural bupivacaine, intramuscular meperidine with haloperidol, synthetic intravenous oxytocin, and prostaglandins such as dinoprostone vaginally, and on the other hand, with suffered pathologies during the first year of life. Cross-sectional retrospective study was carried out on 111 children who attended dental check-ups. Oral examination was carried out to determine MIH involvement. Data on the administration of medications during delivery and the illnesses suffered by the children in the first year of life were taken from the hospital records. Significant relationship with Pearson's chi-square was found between the presence of MIH and the administration of meperidine with haloperidol intramuscularly and the vaginal administration of dinoprostone during labour. Also in children who have suffered serious infections and those who have received antibiotics in early childhood. In recent years there has been a growing trend in many countries to medicalize childbirth even above what the World Health Organization recommends. Some of the drugs used in these protocols could be involved in the appearance of dental mineralization alterations of the MIH type and this would help to explain the increase in its prevalence.

## Introduction

Molar incisor hypomineralization (MIH) is an anomaly of dental development that is associated with causes of systemic origin, with the involvement of one to four permanent first molars and often permanent incisors. Affected teeth have delimited white to brown opacities, porous in texture, and often highly sensitive to thermal changes^1^.

The enamel is weakened and when the teeth come into contact during chewing it tends to give way, producing cracks and cavities. These teeth have great difficulty being anesthetized and have problems with the adhesion of the restorative materials. Also, there is a greater risk of pulp complications that can compromise the viability of the tooth, by presenting incomplete root development between 6 and 10 years old^2^.

In recent years, the prevalence has been increasing^3,4,5,6^, although at the international level the percentages vary greatly between different countries, ranging from 2.4% in Bulgaria, 5.6% in Germany, up to 40.2% in Brazil or 44% in Sydney, affecting both sexes equally^7^.

Prevalence of MIH has increased in recent years, in countries where there are also a high number of inductions to labour.

Etiology of this pathology remains unclear, and it is likely a multifactorial cause, with a higher prevalence in children whose mothers had complications during pregnancy and delivery. At present, both pregnancy and especially childbirth have become frequently medicated processes according to various protocols.

Butera et al. observed a higher prevalence in children whose mothers had complications during pregnancy and delivery^8^. For Pitiphat et al. there is a statistically significant relationship with cesarean delivery, complications during vaginal delivery, and also with serious or chronic diseases in children under 3 years of age^9^. In the study carried out by Koruyucu et al., they showed a significant association of MIH with problems during pregnancy, chickenpox at 4 years of age, and with amoxicillin treatments at an early age^10^. On the other hand, among the factors that have been shown to cause enamel defects in animal experiments are: high fever, hypoxia, hypocalcemia, exposure to antibiotics, and dioxin^11^.

Regarding the pathogenesis, it has been said that of the three stages into which amelogenesis, secretion, transition, and maturation are divided, it would be in the latter that the alteration would most likely occur. It is in the maturation stage where the ameloblasts regulate the final mineralization of the enamel, hardening it as the crystals grow in width and thickness, this layer becoming the one that contains 95% by weight of mineral material. It is in the initial phase of this stage that ameloblasts are especially sensitive to environmental disruptors^12^.

The first permanent molars show their first signs of mineralization at the tips of the cusps, around or shortly after birth^11^. In this sense, it must be considered that some factors related to childbirth, not studied to date, could also influence the appearance of this alteration.

In recent years, cesarean section rates have increased in a generalized way, both in developed and developing countries^13^ without implying a decrease in maternal, neonatal, and infant mortality^14^. The World Health Organization panel of experts proposed that the caesarean section rate should not exceed 10–15% of births, and yet the average number of caesarean sections in Europe in 2010 was 25.2%, Italy being the one with the highest percentage with 38% of births and Iceland the only country within the range established by the WHO with 14.8%.

In vaginal deliveries, the induction rate of labour in Europe ranges from 0.5% in Romania to 16.4% in Ireland, with Spain being the second country in Europe with the second-highest number of labour inducted vaginal deliveries with 15.1%. While the prevalence of MIH in Romania is 14.54%^15^, in the United Kingdom it is 15.9%^16^ and in Spain it is 24.2%^5^.

Induction of labour has also increased in recent years, although both the World Health Organization and the Ministry of Health recommend not exceeding the induction rate of labour of 10%. Spain is characterized by particularly interventional delivery care compared to its European neighbors with a rate of 32%, only surpassed by Belgium with 33%^17^.

Induction of labour is performed with the help of intravenous synthetic oxytocin to increase uterine contractions, also with vaginal prostaglandins that are effective in maturing the cervix and initiating labour, or both. The causes for induction of labour are: maternal arterial hypertension, premature rupture of membranes, oligohydramnios, prolonged pregnancy, delayed intrauterine fetal growth, stillbirth, and interruption of pregnancy, among others, but this practice has become widespread today^18^.

To relieve pain during labour, analgesics and anesthetics are used. The latter include narcotics such as butorphanol, meperidine, fentanyl, and nalpuffin. Narcotics can be administered intramuscularly in the thigh or gluteal, or intravenously, and are usually applied at the onset of labour^19^.

The objective of this study was to assess the possible relationship between the presence of MIH with medications frequently used in childbirth and with pathologies suffered during the first year of life.

## Methods

During 12 months, 111 children between 6 and 9 years of age who attended a dental clinic in the city of La Unión (Murcia) for examination were reviewed. To obtain the sample size, we set ourselves the objective of observing at least a 30% difference in diseases in the first year of life and the study groups. In this way, we obtained that a power of 80% and a significance level of 5%, we should take 36 patients in each group^20^. The study includes those who met the following inclusion criteria; born at the Santa Lucía University General Hospital in Cartagena and early mixed dentition stage; eruption of the lower incisors and first permanent molars^21^.

Previously, the approval of the data protection department of the University of Murcia was obtained and the favorable report of the Ethical Committee of Clinical Research of the Hospital (E.O. 2019-11) and the University (ID: 2291/2019). Informed consent was obtained from all participants and their legal guardians, both of them, in accordance with the Declaration of Helsinki. The examiner's calibration was carried out through a theoretical diagnostic training phase, with training in diagnostic criteria, and then a practical calibration phase with the use of images of previously diagnosed lesions, to observe the concordance.

To determine the degree of MIH involvement, the diagnostic criteria of Ghanim et al.^22^ modified with the observation of the second primary molars. The data were collected in an examination sheet designed for this purpose (“Appendix 1”).

The oral examination was carried out in a dental chair with artificial light, with the help of a mirror and gauze to dry.

A survey was done on the mothers regarding their pregnancy-related details and the infectious diseases that their children could have in the first years of life.

To retrieve the information regarding the medications administered during delivery, and the illnesses of the child in the first year of life, the Hospital's medical records were accessed.

The data were coded and subjected to a pseudonymisation process to ensure the anonymity of the study participants.

The variables to study constituted two groups:

- Diseases of the child in the first year of life: Otitis, respiratory diseases such as bronchitis and bronchiolitis, serious infectious diseases, such as chickenpox, measles, or whooping cough, diseases that caused a high fever, tonsillitis, diarrhea, urine infection, and diseases that required administration of antibiotics.
- Medication administered during childbirth: lytic mixture (meperidine with haloperidol), epidural (bupivacaine, fentanyl, and adrenaline), vaginal dinoprostone, intravenous synthetic oxytocin, and maternal serum antibiotic.

Pearson's chi-square statistical test was used to analyze the data, taking *p* value minor than 0.05 as a significant value.

## Results

Of the 111 children reviewed, 12 who we could not access their birth history, 14 who had been born in other hospitals and 6 who had not yet erupted with permanent incisors and molars were excluded, remaining a sample of 79 participants.

According to this study, the prevalence of MIH involvement is 54.43%. Of the 79 children reviewed, 43 presented MIH to a greater or lesser degree of severity. 79 children completed the first year of life disease questionnaire. Patients who presented MIH in temporary molars also did so in permanent molars, if both had erupted. Those who had not erupted the permanent ones we could not associate it yet. The results can be seen in Table 2.

In the medical records of the hospital, some were incomplete, 20 of them did not specify if the lithic mixture had been applied or not, in 2 it was not seen if they have received epidurals or not, in 22 of them they did not indicate whether they had been administered dinoprostone or not, in 4 they did not talk about the use of oxytocin. The results can be seen in Table 1.

**Table 1:** Medicines administered to the mother during labour.

Variable (*N* = 79)	MIH-affected (N)	%	Non-affected (N)	%	Pearson Chi-square	*P*-value
**Lytic Mi**						
No data	13	65	7	35	21.22	0.000
Yes	16	100	0	0		
No	14	32.6	29	67.4		
**Epidural**						
No data	0	0	2	100	1.417	0.234
Yes	32	60.4	21	39.6		
No	11	45.8	13	54.2		
**Dinoprostone**						
No data	10	45.45	12	54.55	23.225	0.000
Yes	24	92.3	2	7.7		
No	9	29	22	71		
**Oxytocin**						
No data	2	50	2	50	2.591	0.107
Yes	28	62.2	17	37.8		
No	13	43.3	17	56.7		
**Antibiotic**						
Yes	15	60	10	40	0.457	0.499
No	28	51.9	26	48.1		

In relation to the medications administered in childbirth, MIH was significantly related to the lytic mixture, which consists of a half ampoule of meperidine with a half ampoule of haloperidol intramuscularly and with the vaginal administration of dinoprostone, in both cases with values < 0.005 in Pearson's chi-square test (Table 1).

Regarding diseases suffered in the first year of life, significant differences in children who had suffered serious infections in the first year of life were seen, such as whooping cough, measles, or chickenpox. We also observed significant differences (*p* < 0.05) when the child was administered antibiotics during the first year of life (Table 2).

**Table 2:** Diseases suffered during the first year of life.

Variable (N = 79)	MIH-affected (N)	%	Non-affected (N)	%	Pearson Chi-square	*P*-value
**High fever**						
Yes	5	55.6	4	44.4	0.005	0.943
No	38	54.3	32	45.7		
**Serious infections**						
Yes	6	100	0	0	5.436	**0.020**
No	37	50.7	36	49.3		
**Diarrhea**						
Yes	5	55.6	4	44.4	0.005	0.943
No	38	54.3	32	45.7		
**Urine infection**						
Yes	4	66.7	2	33.3	0.392	0.531
No	39	53.4	34	46.6		
**Otitis**						
Yes	8	80	2	20	3.018	0.082
No	35	50.7	34	49.3		
**Tonsillitis**						
Yes	1	100	0	0	0.848	0.357
No	42	53.8	36	46.2		
**Bronchitis**						
Yes	19	65.5	10	34.5	2.271	0.132
No	24	48	26	52		
**Antibiotic**						
Yes	16	72.7	6	27.3	4.115	**0.042**
No	27	47.4	30	52.6		

## Discussion

At present, deliveries are increasingly medicated and instrumented^17^, for this reason, we have considered it important to study what medications had been administered to both children and mothers at the time of delivery since the first permanent molars show their first signs of mineralization at the tips of the cusps around or shortly after birth^11^.

The drugs most frequently used in labour induction have been studied, as these procedures have increased in recent years^17^. To induce labour, synthetic oxytocin is mainly used intravenously to increase uterine contractions and/or prostaglandins when the cervix is still immature, among the prostaglandins are misoprostol and dinoprostone^18^. Misoprostol is a prostaglandin E1 analog initially indicated for the treatment of peptic ulcer^23,24^ but in the 1970s it was found to be effective in inducing cervico-uterine changes, and it began to be used for the induction of labour or in the induction of missed abortion or in the same way for the early termination of pregnancy. Its use in gynecology was “of the label” until it was accepted by the US Food and Drug Administration (FDA) in 2003^25^. Misoprostol can be applied both orally and vaginally, but it has been seen that when applied vaginally, nitric oxide is released that enhances the effect of prostaglandins. This release of nitric oxide when applied vaginally occurs only in pregnant women^26^.

Another prostaglandin used in labour induction is dinoprostone, it is an analog of prostaglandin E2 approved by the US FDA in 1992^25^. It is presented as a vaginal tampon containing 10 mg of dinoprostone. Dinoprostone produces a maturation of the cervix during childbirth, going from being a rigid structure to dilate and soften to allow the fetus to pass through the birth canal, thanks to collagenase, which breaks down the structure of collagen^27^. When administered vaginally, a small part is absorbed through the cervix or lymphatic system and most of the dose passes into the bloodstream^28^. In the package insert, we see that it causes an increase in skeletal abnormalities in rats and rabbits^27^.

Significant differences have been obtained in the use of prostaglandins; in this case, dinoprostone applied using a vaginal tampon in 26 patients. Furthermore, it was found that as the sample was enlarged, the differences increased and Pearson's Chi-square decreased until it reached the value of 0.000.

Synthetic oxytocin is an oxytocin hormone that is given intravenously to help stimulate contractions of the uterus during and after delivery to decrease bleeding. In our study, 45 mothers were administered synthetic oxytocin in labour or during dilation, those who had administered oxytocin only in the immediate puerperium after the baby was born were ruled out, and significant results in relation to MIH were not obtained.

Regional analgesia, such as spinal and epidural blocks, is also common in childbirth. Participants in this study were given bupivacaine with fentanyl and, in some cases, with adrenaline as well. 53 participants were administered epidural analgesia, showing no significant relationship with the appearance of MIH.

Narcotics are used to relieve pain in the initial stages of labour. In this case, the narcotic administered is often the lytic mixture that is administered intramuscularly and consists of 100 mg of dolantine (2 ml, whole ampoule) and one ampoule of haloperidol (1 ml = 5 mg). Haloperidol seeks to counteract the hermetic effect of dolantine. This mixture was administered to 16 of our participants, observing significant differences in its relationship with MIH.

Regarding the use of antibiotics in labour, 25 mothers in this study had to be administered intravenous antibiotics for various circumstances. The antibiotics administered were; ampicillin, penicillin, or erythromycin; however, no significant relationship was found with the presence of MIH.

In relation to the diseases suffered in the first years of life, Wogelius et al. found a relationship between bronchitis and bronchiolitis that required the use of inhaled bronchodilators with the appearance of opacities in dental enamel^29^. In the same way, Jälevik and Norén found a relationship between MIH and the use of antibiotics in patients with tonsillitis and recurrent otitis at an early age Jälevik and Garot^30,^^31^ found it relevant to have suffered from pneumonia, high fever, and otitis half. In the present study, we found significant differences in children who had been given antibiotics in the first year of life. On the other hand, six children who presented serious infectious diseases in the first years of life also presented MIH with great enamel affectations. In this case, this study agreed with other authors who also found it significant to have suffered from chickenpox in the early years^10,32^.

Aine et al. relate prematurity and low birth weight with the appearance of MIH^33^. Children born between 35 and 42 weeks of gestation and with birth weights that vary between 2200 and 4700 g, obtaining no significant differences for MIH were studied. Regarding the possible relationship with prolonged breastfeeding due to the effect of dioxins present in breast milk^11^, in the sample studied, no significant difference was observed between children who had not received breastfeeding and children who had breastfed until, in some cases 5 years.

At present, both pregnancy and childbirth have become widely medicated and protocolized processes. According to the World Health Organization recommendations, the percentage of medicalized deliveries should be lower.

The data obtained in this work encourage further study in prospective studies and on larger samples, the relationship between various intrapartum and perinatal factors with the appearance of MIH, taking into account the possibility that there is a combined effect of several factors.

Deliveries in this study where the lytic mixture and prostaglandins are used, a higher percentage of children with MIH were observed. Relationship was seen with the use of antibiotics during the first year of life but not the use of bronchodilators.

There is also no relationship due to a history of high fever, urine infection, diarrhea, tonsillitis or otitis in early childhood, but we do see a relationship between MIH and a serious infectious disease such as chickenpox, measles, or whooping cough.

## References

[1] Almuallem Z, Busuttil-Naudi A (2018). Molar incisor hypomineralisation (Mih): An overview. Br. Dent. J..

[2] Ghanim A, Silva MJ, Elfrink MEC, Lygidakis NA, Mariño RJ, Weerheijm KL (2017). Molar incisor hypomineralisation (MIH) training manual for clinical field surveys and practice. Eur. Arch. Paediatr. Dent..

[3] Zhao D, Dong B, Yu D, Ren Q, Sun Y (2018). The prevalence of molar incisor hypomineralization: evidence from 70 studies. Int. J. Paediatr. Dent..

[4] Garcia-Margarit M, Catalá-Pizarro M, Montiel-Company JM, Almerich-Silla JM (2014). Epidemiologic study of molar-incisor hypomineralization in 8-year-old Spanish children. Int. J. Paediatr. Dent..

[5] Negre-Barber A, Montiel-Company JM, Catalá-Pizarro M, Almerich-Silla JM (2018). Degree of severity of molar incisor hypomineralization and its relation to dental caries. Sci. Rep..

[6] Yannam SD, Amarlal D, Rekha CV (2016). Prevalence of molar incisor hypomineralization in school children aged 8–12 years in Chennai. J. Indian Soc. Pedod. Prev. Dent..

[7] Biondi AM, Cortese SG, Ortolani AM, Ienco M, Argentieri AB (2015). Prevalencia de hipomineralización molar incisiva en niños con y sin demanda de atención. Rev. Asoc. Odontol. Argent..

[8] Butera A, Maiorani C, Morandini A, Simonini M, Morittu S, Barbieri S (2021). Assessment of genetical, pre, peri and post natal risk factors of deciduous molar hypomineralization (Dmh), hypomineralized second primary molar (hspm) and molar incisor hypomineralization (mih): A narrative review. Children.

[9] Pitiphat W, Luangchaichaweng S, Pungchanchaikul P, Angwaravong O, Chansamak N (2014). Factors associated with molar incisor hypomineralization in Thai children. Eur. J. Oral Sci..

[10] Koruyucu M, Özel S, Tuna EB (2018). Prevalence and etiology of molar-incisor hypomineralization (MIH) in the city of Istanbul. J. Dent. Sci..

[11] Mishra A, Pandey RK (2016). Molar incisor hypomineralization: An epidemiological study with prevalence and etiological factors in Indian pediatric population. Int. J. Clin. Pediatr. Dent..

[12] Fatturi AL, Wambier LM, Chibinski AC, da Assunção LRS, Brancher JA, Reis A (2019). A systematic review and meta-analysis of systemic exposure associated with molar incisor hypomineralization. Community Dent. Oral Epidemiol..

[13] Vogel JP, Betrán AP, Vindevoghel N, Souza JP, Torloni MR, Zhang J (2015). Use of the Robson classification to assess caesarean section trends in 21 countries: A secondary analysis of two WHO multicountry surveys. Lancet Glob Heal..

[14] Ye J, Betrán AP, Guerrero Vela M, Souza JP, Zhang J (2014). Searching for the optimal rate of medically necessary cesarean delivery. Birth..

[15] Savin C, Balan A, Vasilca-Gavrila L, Istrati E, Murariu A (2017). Mih syndrome: Perceptions and knowledge of a sample of dentists From Iasi County. Rom. J. Oral Rehabil..

[16] Balmer R, Toumba J, Godson J, Duggal M (2012). The prevalence of molar incisor hypomineralisation in Northern England and its relationship to socioeconomic status and water fluoridation. Int. J. Paediatr. Dent..

[17] Zeitlin, J. *et al.* European Perinatal Health Report: Health and Care of Pregnant Women and Babies in Europe in 2010. http://hdl.handle.net/2013/ULB-DIPOT:oai:dipot.ulb.ac.be:2013/193256 (2013). 10.1136/jech.2009.087296 19679713

[18] Elzein R, Chouery E, Abdel-Sater F, Bacho R, Ayoub F (2021). Molar–incisor hypomineralisation in Lebanon: Association with prenatal, natal and postnatal factors. Eur. Arch. Paediatr. Dent. [Internet]..

[19] Resnik R, Lockwood CJ, Moore TR, Greene MF, Copel JA, Silver RME (2019). Creasy and Resnik’s Maternal–Fetal Medicine-Principles and Practice.

[20] Whatling R, Fearne JM (2008). Molar incisor hypomineralization: A study of aetiological factors in a group of UK children. Int. J. Paediatr. Dent..

[21] American Academy of Pediatric Dentistry. Management of the developing dentition and occlusion in pediatric dentistry. Chicago III; 408–25 (2021).

[22] Ghanim A, Elfrink M, Weerheijm K, Mariño R, Manton D (2015). A practical method for use in epidemiological studies on enamel hypomineralisation. Eur. Arch. Paediatr. Dent..

[23] Bakker R, Pierce S, Myers D (2017). The role of prostaglandins E1 and E2, dinoprostone, and misoprostol in cervical ripening and the induction of labor: A mechanistic approach. Arch. Gynecol. Obstet..

[24] Vannuccini S, Bocchi C, Severi FM, Challis JRPF (2016). Endocrinology of human parturition. Ann Endocrinol..

[25] FLASOG: Manual de uso de misoprostol en Obstetricia y Ginecología (3th ed), 60–64 (2013).

[26] Väisänen-Tommiska M, Mikkola TS, Ylikorkala O (2005). Misoprostol induces cervical nitric oxide release in pregnant, but not in nonpregnant, women. Am. J. Obstet. Gynecol..

[27] Agencia Española de Medicamentos y Productos Sanitarios. Propess 10mg. https://cima.aemps.es/cima/dochtml/p/62088/P_62088.html.

[28] Esteves-Pereira AP, da Cunha AJLA, Nakamura-Pereira M, Moreira ME, Domingues RM (2021). Twin pregnancy and perinatal outcomes: Data from ‘Birth in Brazil Study’. PLoS One.

[29] Wogelius P, Haubek D, Nechifor A, Nørgaard M, Tvedebrink T, Poulsen S (2010). Association between use of asthma drugs and prevalence of demarcated opacities in permanent first molars in 6-to-8-year-old Danish children. Community Dent. Oral Epidemiol..

[30] Jälevik B, Norén JG (2010). Enamel hypomineralization of permanent first molars: A morphological study and survey of possible aetiological factors. Int. J. Paediatr. Dent..

[31] Garot E, Rouas P, Somani C, Taylor GD, Wong F, Lygidakis NA (2021). An update of the aetiological factors involved in molar incisor hypomineralisation (MIH): A systematic review and meta-analysis.

[32] Ahmadi R, Ramazani N, Nourinasab R (2012). Molar incisor hypomineralization: A study of prevalence and etiology in a group of Iranian children. Iran. J. Pediatr..

[33] Aine L, Backström MC, Mäki R, Kuusela AL, Koivisto AM, Ikonen RS (2000). Enamel defects in primary and permanent teeth of children born prematurely. J. Oral Pathol. Med..

