# Author Correction: Relationship between molar incisor hypomineralization, intrapartium medication and illnesses in the first year of life

**DOI:** 10.1038/s41598-022-07966-y

**Published:** 2022-03-07

**Authors:** Emilia Acosta, Olga Cortes, Sonia Guzman, Montserrat Catala, Monica Lorente, Julian Jesus Arense

**Affiliations:** 1 Department of Paediatric Dentistry, University of Murcia, Murcia, Spain; 2 Department of Paediatric Dentistry, University of Valencia, Valencia, Spain; 3 Gynaecology and Obstetrics Section, Santa Lucía University General Hospital, Cartagena, Spain; 4 Department of Public Health Sciences, School of Medicine, University of Murcia, Murcia, Spain

Correction to: *Scientific Reports* 10.1038/s41598-022-05628-7, published online 31 January 2022

The original version of this Article contained errors in the Reference List.

The References 3, 17, 19, 21, 25 and 27 were incorrectly given as:

3. Zhao, D., Dong, B., Yu, D. & Ren, Q. Objective methods search strategy search results and included subjects prevalence of MIH subgroup analyzes conflict of interest. *Int. J. Paediatr. Dent.* **28**(2), 2021 (2021).

17. Cans C, Colver A, KrÄgeloh-Mann I, Platt M-J, de la Cruz J, Curran R, et al. Health and Care of Pregnant Women and Babies in Europe in 2010. Eur. Perin. Health Rep. (2010)

19. Resnik, R. et al. *Creasy and Resnik’s Maternal–Fetal Medicine-Principles and Practice* 8th edn, 1408 (Elsevier, 2019).

21. American Academy of Pediatric Dentistry. *Management of the developing dentition and occlusion in pediatric dentistry* 408–25 (The Reference Manual of Pediatric Dentistry, Chicago, 2021).

25. Canaval Erazo H. Manual Flasog uso Misoprostol en ginecobstetricia. 2013.

27. Propess—AEMPS. 1377. 68–70.

The correct references are listed below:

3. Zhao D, Dong B, Yu D, Ren Q & Sun Y. The prevalence of molar incisor hypomineralization: evidence from 70 studies. *Int. J. Paediatr. Dent.* **28** (2), 170–179. 10.1111/ipd.12323 (2018).

17. Zeitlin, J., et al. *European Perinatal Health Report: Health and Care of Pregnant Women and Babies in Europe in 2010*. http://hdl.handle.net/2013/ULB-DIPOT:oai:dipot.ulb.ac.be:2013/193256 (2013).

19. Resnik, R. et al. Creasy and Resnik’s Maternal–Fetal Medicine-Principles and Practice (8th ed) 1408 (Elsevier, 2019).

21. American Academy of Pediatric Dentistry. Management of the developing dentition and occlusion in pediatric dentistry. Chicago III; 408–25 (2021).

25. FLASOG: Manual de uso de misoprostol en Obstetricia y Ginecología (3th ed), 60–64 (2013).

27. Agencia Española de Medicamentos y Productos Sanitarios. Propess 10 mg. https://cima.aemps.es/cima/dochtml/p/62088/P_62088.html.

The original Article has been corrected.

